# Tomato Spotted Wilt Virus NSs Protein Supports Infection and Systemic Movement of a Potyvirus and Is a Symptom Determinant

**DOI:** 10.3390/v10030129

**Published:** 2018-03-14

**Authors:** Hernan Garcia-Ruiz, Sergio M. Gabriel Peralta, Patricia A. Harte-Maxwell

**Affiliations:** Nebraska Center for Virology, Department of Plant Pathology, University of Nebraska-Lincoln, Lincoln, NE 68503, USA; ser_gio96@yahoo.es (S.M.G.P), p.a.hartemaxwell@gmail.com (P.A.H.-M.)

**Keywords:** virus movement, gene silencing, antiviral defense, silencing suppression, virus resistance, NSs, HC-Pro, orthotospovirus, tomato spotted wilt virus, turnip mosaic virus

## Abstract

Plant viruses are inducers and targets of antiviral RNA silencing. To condition susceptibility, most plant viruses encode silencing suppressor proteins that interfere with antiviral RNA silencing. The NSs protein is an RNA silencing suppressor in orthotospoviruses, such as the tomato spotted wilt virus (TSWV). The mechanism of RNA silencing suppression by NSs and its role in virus infection and movement are poorly understood. Here, we cloned and tagged TSWV NSs and expressed it from a GFP-tagged turnip mosaic virus (TuMV-GFP) carrying either a wild-type or suppressor-deficient (AS9) helper component proteinase (HC-Pro). When expressed in cis, NSs restored pathogenicity and promoted systemic infection of suppressor-deficient TuMV-AS9-GFP in *Nicotiana benthamiana* and *Arabidopsis thaliana*. Inactivating mutations were introduced in NSs RNA-binding domain one. A genetic analysis with active and suppressor-deficient NSs, in combination with wild-type and mutant plants lacking essential components of the RNA silencing machinery, showed that the NSs insert is stable when expressed from a potyvirus. NSs can functionally replace potyviral HC-Pro, condition virus susceptibility, and promote systemic infection and symptom development by suppressing antiviral RNA silencing through a mechanism that partially overlaps that of potyviral HC-Pro. The results presented provide new insight into the mechanism of silencing suppression by NSs and its effect on virus infection.

## 1. Introduction

RNA silencing contributes to the regulation of gene expression and the maintenance of genome integrity in eukaryotes. In plants, nematodes, and insects, gene silencing is additionally an inducible, adaptable, specific, potent, and essential defense system against virus infection (antiviral RNA silencing) [1,2,3,4,5,6,7]. Both endogenous and antiviral RNA silencing initiate with the processing of double-stranded RNA by Dicer-like (DCL) proteins to form small RNAs of 21 to 24 nt that associate with Argonaute (AGO) proteins to guide slicing or translational repression of target RNAs [2,7,8,9,10,11,12,13]. Sources of viral RNA that might trigger silencing include replication intermediates and self-complementary sequences in viral RNA, yielding primary virus-derived small interfering RNAs (siRNAs) that are necessary but not sufficient to restrict plant virus infection [1]. Restriction of plant virus infection requires silencing amplification by cellular RNA-dependent RNA polymerases (RDR) to establish an antiviral state [3,9,10].

To replicate and establish local and systemic infection, most plant and insect viruses encode silencing suppressor proteins that interfere with both endogenous and antiviral RNA silencing [14]. Mechanisms used by viral suppressors to interfere with RNA silencing include the sequestration of virus-derived and cellular siRNAs, including microRNAs (miRNAs) [2,15,16,17], interaction with and degradation of core components of the RNA silencing pathway, such as DCL proteins [18], AGO proteins [19,20], RDR6, and suppressor of gene silencing 3 (SGS3) [21]. These effects prevent the formation of virus-derived and host siRNAs, their association with AGO proteins, AGO-siRNA targeting of RNA, or RNA silencing amplification. Additionally, potato virus A helper component proteinase (HC-Pro) inhibits RNA silencing by relieving viral RNA translational repression and by disruption of the methionine cycle [22].

Several aspects of plant development are regulated by miRNAs and secondary siRNAs. Both miRNAs and siRNAs downregulate their targets in association with AGO proteins [23,24,25]. While miRNAs are formed from transcript RNA by DCL1, secondary siRNAs are formed by an amplification mechanism that requires RDR6 and SGS3 and is triggered by specialized miRNAs [25,26,27]. Thus, silencing suppressors are determinants of symptom development in virus-infected plants, in part by sequestering miRNAs and secondary siRNAs and by interfering with their biogenesis or activity [15,28]. 

Antiviral RNA silencing is mediated by virus-derived siRNAs, which in association with AGO proteins, target viral RNA for slicing or translational repression [2,11,13]. After initiation, antiviral RNA silencing is amplified by cellular RDR [3,9,10]. Viral silencing suppressors interfere with antiviral RNA silencing, enhancing virus accumulation at the cellular level and promoting cell-to-cell and systemic movement [2,3,8,29,30]. Accordingly, viral suppressors of RNA silencing are pathogenic factors and promote virus infection, movement, and virus accumulation in infected tissue, and regulate host range and tissue specificity [2,18,30,31,32].

Protein NSs is an RNA silencing suppressor encoded by viruses in the genus *Orthotospovirus* (family *Tospoviridae*) [33,34,35]. In tomato spotted wilt virus (TSWV), the type member of the genus *Orthotospovirus*, NSs is a multifunctional protein necessary for systemic movement in plants [36], for the establishment of persistent infection of and transmission by flower thrips (*Frankliniella occidentalis*) [36,37], and is an activator of the hypersensitive response in resistant plants [38,39]. 

TSWV has a wide host range that includes over 1300 plant species [40]. Interestingly, in the absence of silencing suppression by NSs, TSWV loses the ability to infect pepper plants [38,39], highlighting the importance of NSs in virus pathogenicity and host range. Sequence analysis and computational modeling suggest that orthotospoviral NSs has a WG/GW motif and three RNA-binding domains [38] and that NSs suppresses RNA silencing by several mechanisms, including interaction with AGO proteins and binding long double-stranded RNAs and small RNAs [38,41]. The NSs of several orthotospoviruses suppresses silencing of a GFP transgene and interferes with the spread of the systemic RNA silencing signal [33,34]. However, the mechanism of RNA silencing suppression by orthotospoviral NSs and its role in virus infection and movement have not been determined. The lack of an infectious TSWV clone and the lack of a genetically tractable host plant have been limiting factors. Here, we describe a genetically tractable system involving *Nicotiana benthamiana* and *Arabidopsis thaliana* plants and a chimeric potyvirus infectious clone.

When expressed in trans using a standard assay for local antiviral silencing [29], TSWV NSs supports cell-to-cell movement of suppressor-deficient turnip mosaic virus (TuMV, a potyvirus) and turnip crinkle virus (TCV, a carmovirus) [8]. To gain insight into the mechanisms of antiviral RNA silencing suppression by orthotospoviral NSs and determine its role in virus pathogenicity, we cloned and tagged NSs and engineered GFP-tagged TuMV-GFP harboring a wild-type or suppressor-deficient HC-Pro [3] to express NSs, creating recombinant viruses expressing one or two RNA silencing suppressors. 

TSWV NSs mutations in the RNA-binding domain one, which consists of amino acids 45 to 57 (QLYSDSRSKSSFG), abolish silencing suppression and induction of the hypersensitive response [38], emphasizing the importance of the NSs RNA-binding domain one in virus infection. Here, we made a suppression-defective form of NSs by mutating the RNA-binding domain one and expressed it in a potyvirus vector. Genetic stability and pathogenicity of the recombinant viruses were determined in *N. benthamiana* and *A. thaliana* plants. In both host species, chimeric TuMV-GFP clones retained the NSs insert in systemically infected tissue.

Silencing suppression-active NSs but not suppressor-deficient mutant NSs restored pathogenicity to suppressor-deficient TuMV-AS9-GFP. Symptoms developed by systemically infected *N. benthamiana* leaves consisted of a spotted green and yellow mosaic similar to that caused by TSWV. Using *A. thaliana* plants lacking core components of the RNA silencing machinery [2,3], a genetic analysis showed that NSs is necessary and sufficient to overcome defense mechanisms dependent on DCL, RDR, and AGO proteins, namely antiviral RNA silencing. Collectively, the results described here show that NSs can functionally replace potyviral HC-Pro and support the establishment of local and systemic infection and symptom development by a suppressor-deficient potyvirus. The NSs silencing suppression mechanism overlaps with but is not identical to that of potyviral HC-Pro. 

## 2. Materials and Methods 

### 2.1. DNA Plasmids

Standard cloning techniques were used to make all constructs used in this study, gateway entry (pENTR) and destination (pMDC32) vectors [42]. Inserts between the 35S promoter and nopaline synthase (NOS) terminator were verified by Sanger sequencing. The sequence of the oligonucleotide primers is in the 5′ to 3′ orientation.

Plasmid pPZP-ssGFP carrying a single-stranded GFP [29] and pMDC32-NSs carrying TSWV NSs [8] have been described. Plasmids carrying GFP-tagged TuMV (pCB302-TuMV-GFP) and suppressor-deficient TuMV-AS9-GFP (pCB302-TuMV-GFP) have been described [3].

pMDC32-Empty. An empty vector was made by removing the AscI-PacI fragment from pMDC32.

pMDC32-p19-HA. HA-tagged protein p19 from tomato bushy stunt virus in pCB302-p19-HA [15] was moved into pMDC32. Using oligos p19ORF-F (caccATGGAACGAGCTATACAAGG) and p19-BglII-R (AGATCTAGATTAAGCGTAGTCTGGG), the p19 ORF and the HA tag from pCB302-p19-HA [15] were amplified by PCR, cloned into pENTR, and moved into pMDC32 by LR recombination.

pMDC32-NSs-6HIS3xFLAG. The 6HIS3xFlag tag was added to the C terminus of NSs by PCR amplification of the NSs ORF from pMDC32-NSs [8] with primers NSs-ORF-F (caccATGTCTTCAAGTGTTTATGAGTC) and 6xHIS3xFLAG-NSs (ggcggccgctctagaTCACTTGTCATCGTCATCCTTGTAGTCGATGTCATGATCTTTATAATCACCGTCATGGTCTTTGTAGTCGTGATGGTGATGGTGATGTTTTGATCCTGAAGC). The PCR product was inserted into pENTR by TOPO cloning to generate pENTR-NSs-6HIS3xFLAG and moved into pMDC32-NSs-6HIS3xFLAG by LR recombination. 

pMDC32-NSs-S48A-R51A-6HIS3xFLAG. Inactivating mutations S48A and R51A in NSs RNA-binding domain one [38] were introduced through site-directed mutagenesis by rolling circle PCR [43] using pENTR-NSs-6HIS3xFLAG as the template and the primers NSs-S48A-R51A (gCaGAtTCAgctAGCAAAAGTAGCTTTGGC) and NSs-R (ATACAGCTGGGTTTGAACTAGTGGAGAACC) to generate pENTR-NSs-S48A-R51A-6HIS3xFLAG, and moved into pMDC32 as described above.

pMDC32-NSs-3-6HIS3xFLAG. Inactivating mutations K182A (NSs-1) in the GKT motif and L413A (NSs-2) in the YL motif [44] were introduced by site-directed mutagenesis by rolling circle PCR [43] in two parts. The K182A mutation was introduced using pENTR-NSs-6HIS3xFLAG as the template and the primers NSs-1-F (gctGTGAATGTTCTATCCCCTAACAG) and NSs-1-R (GCCTAAAGCTTGATTGTAGCACATCTCG) to generate pENTR-NSs-1-6HIS3xFLAG. The L413A (NSs-2) mutation was introduced using pENTR-NSs-1-6HIS3xFLAG as the template and the primers NSs-2-F (gctGACAGCATCCAAATCCC) and NSs-2-R (GTAAGACATAGTTTGTGTGTTAGATGG) to generate pENTR-NSs-3-6HIS3xFLAG and, by LR recombination, pMDC32-NSs-3-6HIS3xFLAG.

pENTR-NIb-CP (TuMV). This partial TuMV clone carries part of NIb, CP, the 3′ UTR, and the vector sequences corresponding to nt 8516 to 9916 in pCB302-Wt-TuMV [3]. A PCR product was generated using pCB302-Wt-TuMV [3] and template and oligos TuMV-Nib-ENTR (caccGCGATGATTGAGTCGTGGGG) and pCB302-Ter-Rev (ATCGCAAGACCGGCAACAGG) and moved into pENTR by TOPO cloning [42].

pENTR-NIb-NSsHF-CP (TuMV). 6HIS-3xFLAG-tagged NSs (NSs-HF) was introduced in frame between NIb and CP by overlapping PCR. Overlapping fragments A, B, and C were generated as follows: (A) pENTR-NIb-CP as template and primers TuMV-Nib-ENTR and Nib-NSs-R (GTCTGAATGATCGACTCATAAACACTTGAAGAtgCctggtgataaacacaagcctcagc); (B) pENTR-NSs-6HIS3xFLAG as template and primers Nib-NSs-F (gctgaggcttgtgtttatcaccagGcaTCTTCAAGTGTTTATGAGTCGATCATTCAGAC) and Flag-CP-R (cctgcatcaagcgtttcacctgcCTGGTGATAGACACACTTGTCATCGTCATCCTTGTAGTCG). (C) pENTR-NIb-CP as template and primers Flag-CP-F (CGACTACAAGGATGACGATGACAAGTGTGTCTATCACCAGgcaggtgaaacgcttgatgcagg) and pCB302-Ter-Rev. Individual fragments A, B, and C were stitched and amplified with oligos TuMV-Nib-ENTR and pCB302-Ter-Rev and moved into pENTR by TOPO cloning. M was changed to A in NSs to complete an NIa cleavage site at the N terminus of NSs and an extra NIa cleavage site was added between NSs-HF and the coat protein.

pENTR-NIb-NSsHF-S48A-R51A-CP (TuMV). Mutant NSsHF-S48A-R51A was introduced in frame between NIb and CP by overlapping PCR, as described above, except that pENTR-NSsHF-S48A-R51A-6HIS3xFLAG was used as the template for making fragment B.

pCB302-TuMV-AS9-GFP-NSs-HF. The 6HIS-3xFLAG-tagged NSs (NSs-HF) was introduced in frame between NIb and CP. In plasmid pCB302-TuMV-AS9-GFP [3], the MluI-PvuI fragment was replaced with that of pENTR-NIb-NSsHF-CP (TuMV).

pCB302-TuMV-AS9-GFP-NSs-S48A-R51A-HF. The mutant NSsHF-S48A-R51A was introduced in frame between NIb and CP. In plasmid pCB302-TuMV-AS9-GFP [3], the MluI-PvuI fragment was replaced with that of pENTR-NIb-NSsHF-S48A-R51A-CP (TuMV). 

pCB302-TuMV-GFP-NSs-HF. To introduce in frame the 6HIS-3xFLAG-tagged NSs (NSs-HF) between NIb and CP in plasmid pCB302-TuMV-GFP [3], the MluI-PvuI fragment was replaced with that of pENTR-NIb-NSsHF-CP (TuMV).

pCB302-TuMV-GFP-NSs-S48A-R51A-HF. To introduce in frame the NSsHF-S48A-R51A between NIb and CP in plasmid pCB302-TuMV-GFP [3], the MluI-PvuI fragment was replaced with that of pENTR-NIb-NSsHF-S48A-R51A-CP (TuMV).

### 2.2. Plant Materials

*A. thaliana* plants used were ecotype Columbia-0 (Col-0), grown at 22 °C under long day conditions (16 h light and 8 h dark). Triple mutant dcl2-1 dcl3-1 dcl4-2 [45] and ago2-1, rdr1-1, and rdr6-15 single mutants [46] were described and characterized [2,3] as before. *N. benthamiana* plants were wild-type and grown at 24 °C under long day conditions.

### 2.3. Agrobacterium Transformation and Agroinfiltration

*Agrobacterium tumefaciens* strain GV3101 was transformed by electroporation as described [8] with pMDC32 plasmids carrying NSs clones or tombusviral p19-HA tagged at the C terminus with the HA epitope [15]. Single-stranded green fluorescent protein (ssGFP) reporter was carried by pPZP-35S-GFP [29]. Parental TuMV-GFP or derivatives were expressed from pCB302 plasmids [2,3].

Using *N. benthamiana* plants at the 5 to 6 leaf stage, a standard assay [47] was used to measure silencing suppression of the ssGFP reporter. *A. tumefaciens* cells carrying the ssGFP (OD600 = 0.25) were infiltrated in combination with NSs (OD600 = 0.5) or controls. Empty pMDC32 and tombusviral p19-HA were used as negative and positive controls, respectively. For each treatment, ten plants were infiltrated at leaves three and four and the experiment was repeated three times. Plants were incubated for four days in a growth chamber at 24 °C with 16 h of light and 8 h of darkness (long day). Photographs of infiltrated leaves were taken under ultraviolet (UV) light at 4 days post infiltration and GFP fluorescence was measured using photographs and the green channel in ImageJ (https://imagej.net/Welcome) [8,33]. At 4 days post infiltration, infiltrated leaf samples were collected for protein extraction as described [8]. 

In *N. benthamiana* and *A. thaliana*, infection by TuMV and derivatives was launched by agroinfiltration [48]. *A. tumefaciens* cells carrying TuMV-GFP or derivatives were re-suspended to an OD600 = 0.05. Cells carrying empty pMDC32 were used as the negative control. At the time of infiltration, *N. benthamiana* plants were at the 5–6 leaf stage and *A. thaliana* plants were 16 days old. Two *N. benthamiana* and four *A. thaliana* leaves were infiltrated per plant, and plants were incubated for 15 days in a growth chamber at 24 °C or 22 °C, respectively, and under long day conditions. At 10 days post inoculation (dpi), leaf samples were collected for protein extraction and analysis. The onset of systemic infection was monitored daily under UV light. The experiment was repeated three times. 

### 2.4. Tomato Spotted Wilt Virus Mechanical Inoculation

The Hawaii isolate of (*Tomato spotted wilt virus*) TSWV was maintained, propagated, and mechanically inoculated as described [8]. 

### 2.5. Western Blotting

Total protein was extracted as described [8] by grinding leaf samples in 0.5 mL glycine buffer [19] per 1 g of leaves. After a Bradford assay, extracts were normalized to 0.5 mg/mL. For Western blotting, 12.5 μg were used and proteins were separated by gel electrophoresis at 150 V for 60 min and transferred to nitrocellulose membranes. GFP was detected with anti-GFP antibody (Merck Millipore, Darmstadt, Germany) at a 1:4000 dilution. 6HIS3xFlag-tagged NSs was detected with anti-Flag-peroxidase antibody (Sigma-Aldrich, St. Louis, MO, USA) at a 1:10,000 dilution. HA-tagged p19 [15] was detected using anti-HA antibody with peroxidase (Roche, Basel, Switzerland) at a 1:1000 dilution. HSP70 was used as the loading control and detected using primary anti-HSP70 (Merck Millipore, Darmstadt, Germany) at 1:6000 dilution and secondary antibody (goat anti-rabbit immunoglobulin G, NA934-1; GE Healthcare, Little Chalfont, UK) at a 1:10,000 dilution. TuMV CP was detected with anti-CP (PVAS-134 at 1:10,000 dilution) with secondary antibody NA934-1 at a 1:10,000 dilution. Primary and peroxidase-conjugated antibodies were incubated at 4 °C overnight and secondary antibodies for 30 min at room temperature. Chemiluminescence was measured with Clarity Western ECL Substrate and a ChemiDoc^®^ MP Imaging System (Bio-Rad, Hercules, CA, USA). 

### 2.6. Confocal Microscopy

Systemically infected *N. benthamiana* leaves and controls were collected at 10 dpi for microscopy. Colorimetric images were obtained using a scanner (Epson V600) with visible light. A Nikon TI-2 microscope with a DS-Qi2 camera was used to image whole leaves for GFP (excitation at 470/40 nm and emission at 525/50 nm), pseudocolored green, and chlorophyll autofluorescence pseudocolored red (excitation 620/60 nm and emission at 700/75 nm). A 2× lens was used to image fluorescent data and the resulting images were stitched together with NIS-Elements (Version 5.02, Nikon Instruments, Inc., Tokyo, Japan).

## 3. Results

### 3.1. NSs Tagging and Mutational Inactivation

**Figure 1:**
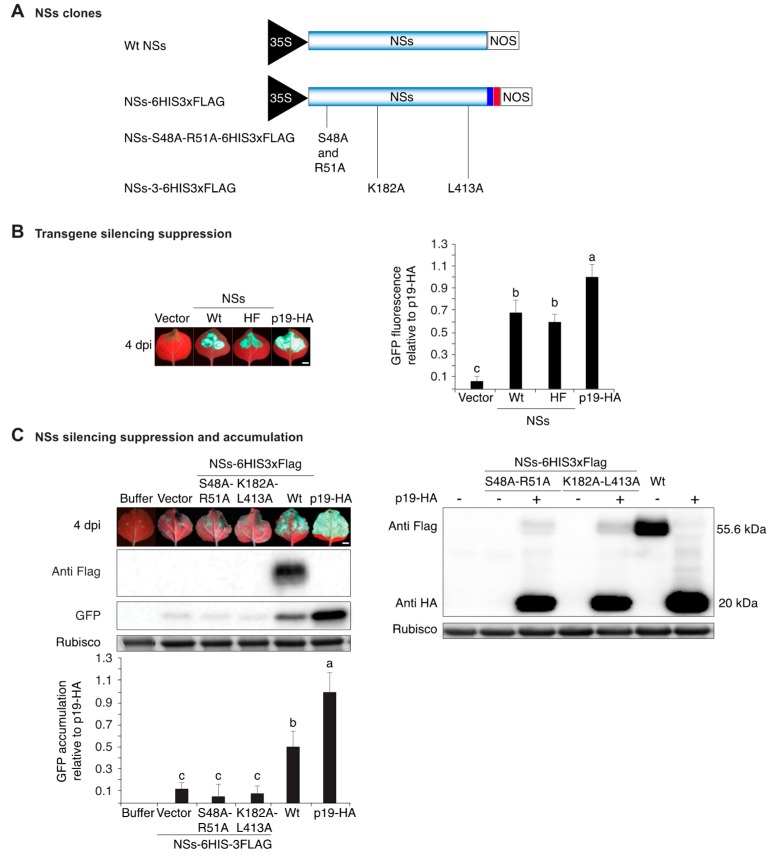
GFP transgene silencing suppression and NSs protein accumulation. (**A**) Representation of TSWV NSs clones placed between the 35S promoter and the nopaline synthase (NOS) terminator in pMDC32. A 6HIS-3xFLAG (referred to as HF) tag added in frame at the C terminus is indicated by colored boxes. Inactivating mutations in RNA binding domain one (S48A and R51A), in the GKT motif and in the YL motif are indicated; (**B**) Suppression of transgene silencing by wt and HF-tagged NSs. ssGFP was infiltrated alone or in combination with NSs in wt *N. benthamiana* leaves. Empty vector and HA-tagged protein p19 from tomato bushi stunt virus (p19-HA) were used as negative and positive controls, respectively. Four days after infiltration, GFP fluorescence was visualized and photographs taken under ultraviolet light. The histogram shows average GFP fluorescence ± standard error (18 leaves from three repetitions) relative to leaves infiltrated with p19-HA; (**C**) Silencing suppression and accumulation of NSs-HF proteins. The experiment was as in (**B**) plus a control infiltrated with buffer. Representative western blots showing NSs-HF and GFP accumulation. Rubisco was used as loading control. The histogram shows average GFP accumulation ± standard error (four biological replicates) relative to that observed in leaves infiltrated with p19-HA. The blot on the right shows NSs-HF accumulation in the presence (+) and absence (−) of p19-HA. In (**B**,**C**) bars with the same letter are not significantly different (Tukey’s test with α = 0.05). Scale bars: 1 cm.

A 6HIS3xFlag tag (HF) was added at the C terminus of TSWV NSs (NSs-HF) and expressed from the 35S promoter in pMDC32 [42] (Figure 1A). Silencing suppression was tested using a single-stranded GFP transgene in wt *N. benthamiana* following a standard assay [47]. At 4 days post infiltration, pictures were taken under UV light and GFP fluorescence was estimated using the green channel in ImageJ [8,33]. GFP fluorescence in the presence of NSs-HF was as bright as in the presence of wt NSs. Consistent with previous results for TSWV NSs [34], for both NSs and NSs-HF, GFP fluorescence was 60% ± 10% of that observed in the presence of HA-tagged tombusviral p19 (p19-HA) [15] used as the positive control (Figure 1B). Thus, the 6HIS3xFlag tag did not affect transgene silencing suppression activity of NSs. 

In TSWV, the NSs RNA-binding domain one is necessary for transgene silencing suppression, induction of the hypersensitive response [38], and consists of amino acids 45 to 57 [38]. Similarly, the GKT motif and the YL motif are necessary for transgene silencing suppression [44]. To inactivate NSs-HF, S48A, and R51A, mutations were introduced into the RNA-binding domain one [38]. In a second clone, inactivating mutations K182A (NSs-1) and L413A (NSs-2) were introduced in the GKT motif and in the YL motif (NSs-3) [44], respectively (Figure 1A). Transient assays, as described above, showed that both mutants lost transgene silencing suppression activity (Figure 1C). Thus, herein these clones are referred to as suppressor-deficient NSs-HF. In contrast, wt NSs-HF suppressed silencing and GFP protein accumulated to 50% ± 12% of that observed in the presence of p19-HA (Figure 1C). Using an anti-Flag antibody, NSs-HF was detected in infiltrated leaves. In contrast, suppression-deficient NSs-HF mutants were below detection (Figure 1C). To verify the expression of suppressor deficient NSs-HF clones, their accumulation was determined in the presence and in the absence of p19-HA. Mutant NSs-HF proteins were only detected in the presence of p19-HA and accumulated to only 8% of wt NSs-HF (Figure 1C). Low accumulation of suppressor-deficient mutant NSs-HF was due to reduced protein stability (see below). 

### 3.2. NSs Supports Systemic Movement of Suppressor-Deficient TuMV-AS9-GFP 

**Figure 2:**
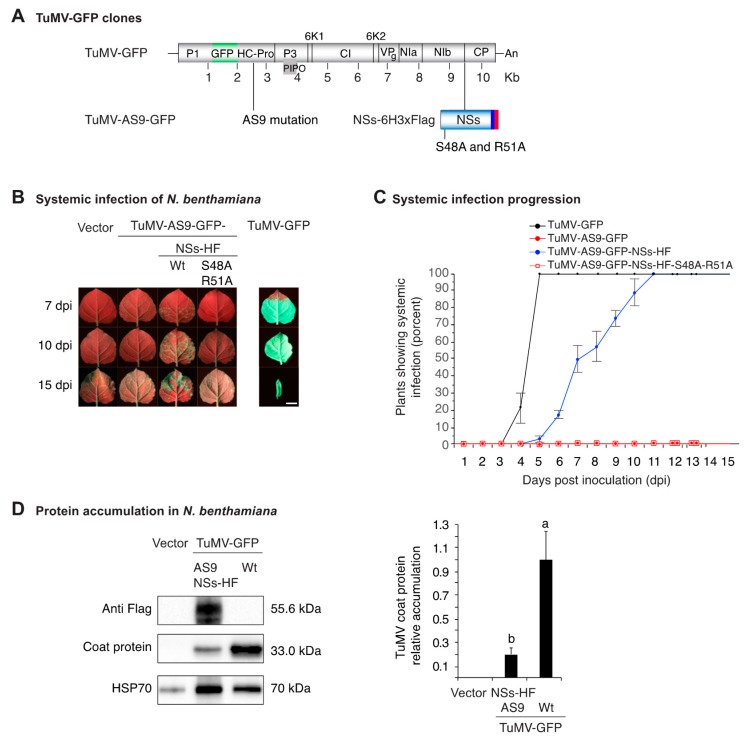
Pathogenicity in *N. benthamiana* and accumulation of suppressor-deficient TuMV-AS9-GFP derivatives expressing NSs-HF. (**A**) Representation of TuMV-GFP clones showing insertion of GFP between P1 and HC-Pro, the AS9 mutation on HC-Pro, and insertion of NSs-HF between NIb and the coat protein. An NIa cleavage site (CVYHQ/A) was added between NIb and NSs-HF and between NSs-HF and the coat protein to promote release of NSs-HF after polyprotein processing. Inactivating mutations on NSs RNA binding motif one are indicated. (**B**) Visualization under UV light of systemic infection in leaves at 7, 10 and 15 dpi. TuMV-GFP is shown for comparison. Scale bar: 2 cm. (**C**) Proportion (%) of plants showing systemic infection as determined by UV illumination. Points represent average (±standard error) of three repetitions, each consisting of 12 plants per treatment. (**D**) Virus accumulation in systemically infected leaves at 10 dpi. Representative western blots showing NSs-HF and coat protein accumulation in the same sample. HSP70 was used as a loading control. The blot was cut in three parts before antibody probing. The histogram shows average coat protein accumulation ± standard error (four biological replicates) relative to TuMV-GFP. Bars with the same letter are not significantly different (Tukey’s test with α = 0.05).

**Figure 3:**
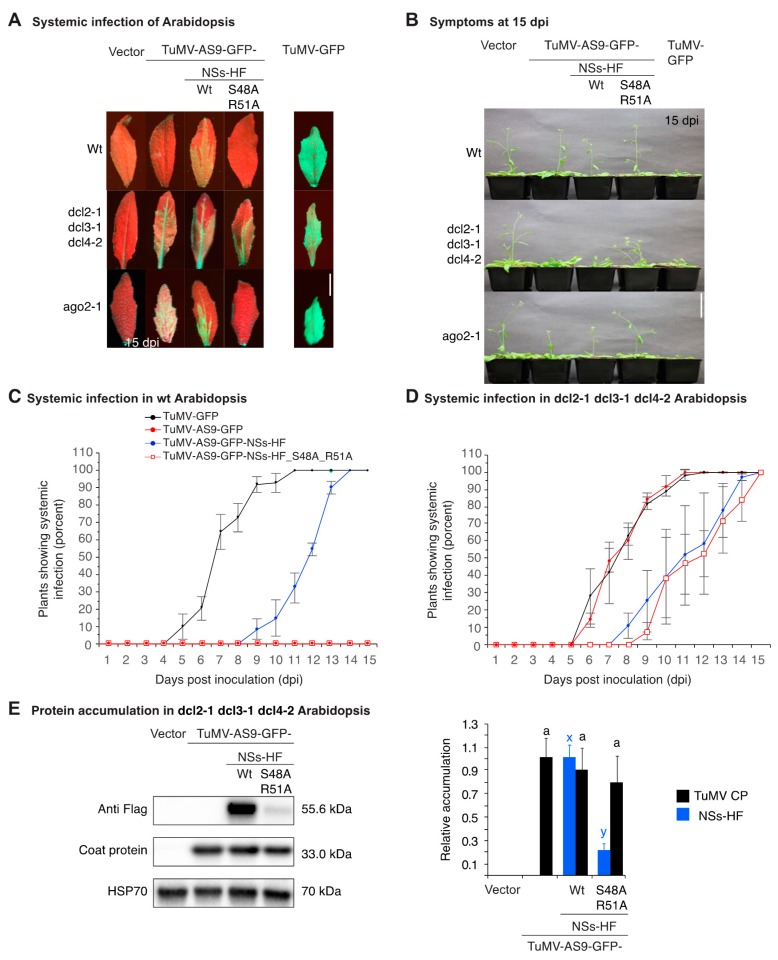
Pathogenicity and accumulation in *A. thaliana* (Arabidopsis). Wt, dcl2-1 dcl3-1 dcl4-2 triple, and dcl4-2 single mutant plants were inoculated by agroinfiltration with TuMV-AS9-GFP derivatives expressing NSs-HF, empty vector, TuMV-AS9-GFP or TuMV-GFP. (**A**) Visualization under UV light of systemic infection in cauline leaves at 15 dpi. Scale bar: 1 cm. (**B**) Representative plants showing symptoms at 15 dpi. Scale bar: 6 cm. (**C**) Proportion (%) of wt or (**D**) dcl2-1 dcl3-1 dcl4-2 triple mutant plants with systemic infection of inflorescence. Points represent average (±standard error) of three repetitions, each consisting of 12 plants per treatment. (**E**) Representative western blots showing NS-HF and coat protein accumulation at 10 dpi. HSP70 was used as a loading control. The histogram shows average (±standard error) coat protein or NSs-HF accumulation (four biological replicates) relative to TuMV-GFP or to wt NSs-HF, respectively. Bars with the same letter do not significantly differ (Tukey’s test with α = 0.05).

When expressed in trans, NSs-HF suppressed local transgene silencing (Figure 1) and wt NSs suppressed local antiviral RNA silencing, leading to the formation of local infection foci [8]. We hypothesized that NSs supports viral systemic movement. To test this hypothesis, we expressed NSs-HF in cis from TuMV-AS9-GFP, which cannot infect wt *N. benthamiana* or wt *A. thaliana* due to an inactivation mutation (AS9) in the silencing suppressor HC-Pro [3]. NSs-HF or NSs-HF-S48A-R51A with inactivating mutations (Figure 1A) were placed between NIb and the coat protein in a TuMV-AS9-GFP infectious clone (Figure 2A). Virus infection was launched by agroinfiltration [48] in *N. benthamiana* and wt *A. thaliana*. Empty vector and TuMV-AS9-GFP were used as the negative controls while TuMV-GFP [3] was used as a positive control. Virus movement and the establishment of systemic infection was tracked for 15 days based on GFP fluorescence under UV light and confirmed by Western blotting [3]. In both *N. benthamiana* and wt *A. thaliana*, NSs-HF restored pathogenicity to suppressor-deficient TuMV-AS9-GFP (Figure 2B and Figure 3A) and supported systemic virus movement through the entire plant (Table 1). In contrast, chimeric virus expressing suppressor-deficient NSs-HF-S48A-R51A failed to establish local and systemic infection (Figure 2B and Figure 3A,B and Table 1). NSs-HF-S48A-R51A has inactivating mutations in the RNA-binding domain one [38] (Figure 1). These contrasting differences show that silencing the suppression by NSs-HF was necessary and sufficient to restore establishment of infection and promote systemic movement of suppressor-deficient TuMV-AS9-GFP. This effect requires NSs RNA-binding domain one.

In both *N. benthamiana* and wt *A. thaliana*, systemic infection of TuMV-AS9-GFP expressing NSs-HF was slower than that observed for TuMV-GFP (Figure 2B,C and Figure 3C), which could be due to the loss of the NSs insert or to replication or movement defects caused by the presence of NSs-HF in the TuMV genome. To determine the stability of the NSs-HF insert, systemically infected leaf samples were collected from individual *N. benthamiana* plants at 10 dpi and total protein was extracted for Western blotting. Anti-coat protein (CP) and anti-Flag antibodies were used to probe for both TuMV coat protein and NSs-HF, respectively. In all samples, both coat protein and NSs-HF were detected. Thus, the NSs-HF insert was maintained (Figure 2D). Coat protein accumulation of TuMV-AS9-GFP expressing NSs-HF was only 20% of that observed for wt TuMV-GFP (Figure 2D), suggesting a defect caused by the NSs-HF insert.

### 3.3. NSs Promotes Systemic Virus Movement by Suppressing Antiviral RNA Silencing

TuMV-AS9-GFP expressing suppressor-deficient NSs-HF-S48A-R51A failed to establish local and systemic infection in *N. benthamiana* and wt *A. thaliana* (Figure 2B, Figure 3A and Table 1). This could be the result of replication or movement defects, or both, due to the lack of silencing suppression. Viruses lacking a silencing suppressor infect plants that cannot establish antiviral RNA silencing [1,2,3,18,49]. The RNA silencing model predicts that TuMV-AS9-GFP expressing the suppressor-deficient NSs-HF-S48A-R51A would infect plants that are incapable of establishing antiviral RNA silencing. In contrast, if TuMV-AS9-GFP expressing the suppressor-deficient NSs-HF-S48A-R51A is unable to replicate or move, it would not infect plants not able to establish a silencing response. To distinguish these models, we used *A. thaliana* mutants lacking core components of the RNA silencing machinery [2,3]. Chimeric TuMV-AS9-GFP expressing NSs-HF or suppressor-deficient NSs-HF-S48A-R51A were used to inoculate dcl2-1 dcl3-1 dcl4-2 *A. thaliana* triple mutants and rdr1-1, rdr6-15 and ago2-1 single-mutant plants, which are susceptible to suppressor-deficient TuMV-AS9-GFP [2,3]. Parental TuMV-GFP and suppressor-deficient TuMV-AS9-GFP were used as pathogenic and non-pathogenic controls, respectively [3].

Chimeric TuMV-AS9-GFP expressing NSs-HF or suppressor-deficient NSs-HF-S48A-R51A established systemic infection of leaves (Figure 3A) and inflorescence of dcl2-1 dcl3-1 dcl4-2 triple mutants (Table 1). Accordingly, the lack of infection in *N. benthamiana* and wt *A. thaliana* plants by TuMV-AS9-GFP expressing suppressor-deficient NSs-HF-S48A-R51A is due to the lack of silencing suppression activity of the NSs insert (Figure 1C and Figure 3A).

Virus accumulation in systemically infected inflorescence of dcl2-1 dcl3-1 dcl4-2 triple mutants was determined by Western blot in samples collected from individual plants at 15 dpi. Accumulation of TuMV coat protein and NSs-HF in the same sample was determined as described above. Consistent with results obtained in systemically infected *N. benthamiana* (Figure 2D), in all systemically infected *A. thaliana* inflorescence samples, both coat protein and NSs-HF were detected (Figure 3E). Thus, the NSs-HF insert was maintained after systemic infection of *N. benthamiana* and *A. thaliana*. Coat protein accumulated to similar levels for TuMV-AS9-GFP and chimeric viruses expressing NSs-HF or suppressor-deficient NSsHF-S48A-R51A (Figure 3E). However, NSsHF- S48A-R51A protein accumulated to only 20% of NSs-HF. Potyviruses form a polyprotein that is processed by viral proteases to produce the same number of individual mature proteins [50]. Reduced accumulation in the presence of similar amounts of coat protein made from the polyprotein shows that NSsHF-S48A-R51A protein is less stable than NSs-HF. Accordingly, NSsHF-S48A-R51A is both unstable and inactive in silencing suppression, and the RNA-binding domain one determines both features. Several other NSs mutations that abolished silencing suppression also caused reduced protein accumulation [33]. These observations suggest that NSs silencing suppression activity and stability are linked.

In dcl2-1 dcl3-1 dcl4-2 triple mutant plants, TuMV-GFP and suppressor-deficient TuMV-AS9-GFP established systemic infection of inflorescence at a similar rate (Figure 3D). dcl2-1 dcl3-1 dcl4-2 triple mutants cannot establish a silencing response to fight virus infection [3,18,49]. Accordingly, in dcl2-1 dcl3-1 dcl4-2 triple mutants, systemic virus movement is independent of silencing suppression. Systemic movement by TuMV-AS9-GFP was similar when expressing active NSs-HF or suppression-deficient NSs-HF-S48A-R51A (Figure 3D). However, clones carrying an NSs insert established systemic infection slower than those not expressing NSs (Figure 3D). Thus, the NSs insert caused a slow systemic movement phenotype that was independent of silencing suppression. This could be related to alterations in RNA secondary structures, replication, encapsidation efficiency, polyprotein processing, or a combination. In potyviruses, codon usage bias does not explain translational selection [51] and TSWV infects both *N. benthamiana* and *A. thaliana* [52]. Accordingly, changes in codon usage due to the NSs insert are unlikely to affect translation. 

Infection of rdr1-1, rdr6-15, and ago2-1 single-mutant plants by suppressor-deficient TuMV-AS9-GFP is restricted to cauline leaves [2,3], while inflorescence remains uninfected due to the redundant antiviral activity of RDR1 and RDR6 [3], and AGO1 and AGO10 [2]. Interestingly, TuMV-AS9-GFP expressing NSs-HF established systemic infection of inflorescence in rdr1-1, rdr6-15, and ago2-1 single-mutant plants (Table 2), suggesting that NSs interferes with antiviral activity mediated by RDR1, RDR6, AGO1, and AGO10. In contrast, TuMV-AS9-GFP expressing suppressor-deficient NSs-HF-S48A-R51A did not systemically infect rdr1-1, rdr6-15, and ago2-1 single-mutant plants (Figure 3A and Table 2). In virus-infected plants, both the virus and the RNA silencing signal move systemically [53]. Viruses are protected by silencing suppressors that prevent spread of the silencing signal [33,34] or by a combination of mechanisms that prevent targeting of viral RNA by the silencing machinery [2,15,16,17]. TSWV NSs prevents movement of the silencing signal [33,34]. In TuMV-AS9-GFP expressing suppressor-deficient NSs-HF-S48A-R51A, there is no suppressor preventing the spread of the silencing signal or protecting the viral genome from RNA silencing. One model to explain the lack of infection of rdr1-1, rdr6-15, and ago2-1 single mutants by TuMV-AS9-GFP expressing suppressor-deficient NSs-HF-S48A-R51A is that due to the slow movement phenotype, the RNA silencing signal moves faster than the virus and an antiviral state is established ahead of virus infection.

This genetic analysis shows that NSs promotes virus movement and the establishment of systemic infection by suppressing an antiviral defense mechanism that requires DCL, RDR, and AGO proteins. 

### 3.4. NSs Is a Symptom Determinant 

**Figure 4:**
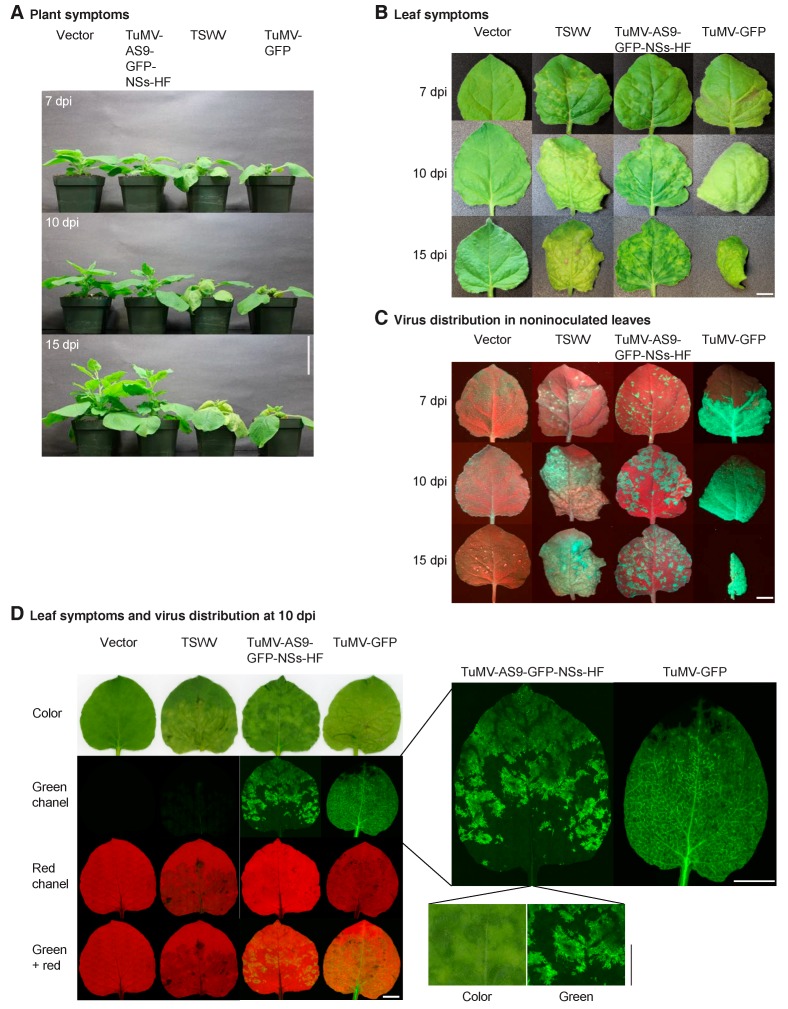
Symptoms and virus distribution in noninoculated leaves of *N. benthamiana* plants. Plants were inoculated by agroinfiltration with empty vector, TuMV-AS9-GFP-NSs-HF, or TuMV-GFP. TSWV infection was initiated by mechanical inoculation. (**A**) Representative plant symptoms at 7, 10 and 15 dpi. Scale bar: 7.5 cm. (**B**) Photographs showing representative noninoculated leaf symptoms in plants described in (**A**). (**C**) Distribution of virus infection determined by GFP fluorescence under UV light for leaves in (**B**). (**D**) Leaf symptoms and distribution of GFP as determined by confocal microscopy using green and red channels. Leaves infected with TuMV-AS9-GFP-NSs-HF or TuMV-GFP are shown at a larger size to illustrate spatial distribution of virus infection in the leaf. An area of the same size was cropped from the color and green image of the leaf infected by TuMV-AS9-GFP-NSs-HF. Chlorotic areas overlap with virus infection. In (**B**–**D**), scale bars: 1 cm.

*N. benthamiana* plants systemically infected by chimeric TuMV-AS9-GFP-NSs-HF showed mild symptoms that correlated with a slow establishment of systemic infection (Figure 2B,C). At 10 dpi, systemically infected leaves showed green and chlorotic spotted mosaic similar to that caused by TSWV [8], and different from TuMV symptoms [3]. These observations suggest that NSs is a symptom determinant. To test this hypothesis, *N. benthamiana* plants were inoculated the same day with TuMV-AS9-GFP, TuMV-AS9-GFP-NSs-HF, TSWV, or TuMV-GFP. Establishment of systemic infection was monitored for 15 dpi. Symptoms induced by TuMV-AS9-GFP-NSs-HF were similar to those induced by TSWV and less severe than those induced by TuMV-GFP (Figure 4A). Symptoms induced by TuMV-GFP were more severe than those induced by TSWV. The difference was particularly clear at 7 dpi and faded at 15 dpi (Figure 4A). 

In plants inoculated with TuMV-AS9-GFP-NSs-HF, systemically infected leaves showed a mosaic of green and chlorotic spots similar to those caused by TSWV, which were not present in plants inoculated with TuMV-GFP (Figure 4B). Examination under UV and visible light showed that infection by TuMV-GFP progressed from the base to the top of the leaf and covered most of the leaf surface by 7 dpi (Figure 2B and Figure 4C). In contrast, TuMV-AS9-GFP-NSs-HF infection was localized to spots and did not cover the entire leaf during the 15 days of the experiment (Figure 2B and Figure 4C). Similarly, infection by TSWV induced a mosaic of green and chlorotic spots. Some of the chlorotic spots had localized necrosis that resulted in autofluorescence under UV light (Figure 4C) that was not due to GFP resulting from infection (Figure 4D). Confocal microscopy analysis of non-inoculated leaves showed that TuMV-AS9-GFP-NSs-HF caused chlorotic spots that overlapped with GFP fluorescence derived from systemic infection, while normal green areas did not show signs of virus infection (Figure 4D). Accordingly, in the mosaic of green and chlorotic spots, TuMV-AS9-GFP-NSs-HF infection induced a change in color in an NSs-dependent manner.

Infection by TuMV-AS9-GFP-NSs-HF was distributed in spots and GFP fluorescence was more intense in the mesophyll than in the veins. In contrast, in plants inoculated with TuMV-GFP, non-inoculated leaves were uniformly covered by virus infection from the bottom to the tip and GFP fluorescence was more intense in the veins than in the mesophyll (Figure 4D). Thus, individually, NSs and HC-Pro determined the pattern of systemic infection and spatial virus distribution in the leaves.

In *A. thaliana*, symptoms caused by TuMV-GFP were more severe than those observed for TuMV-AS9-GFP-NSs-HF (Figure 3B). Symptoms caused by TuMV-AS9-GFP-NSs-HF were more severe than those caused by TuMV-AS9-GFP expressing suppressor-deficient NSs-HF (Figure 3B). Thus, in *A. thaliana*, as in *N. benthamiana*, NSs promoted systemic infection and symptom development. Both effects are dependent on NSs silencing suppression.

These results show that silencing suppression by NSs restored pathogenicity to suppressor-deficient TuMV-AS9-GFP, promoting local, systemic infection, and symptom development following a pattern similar to that of TSWV.

### 3.5. NSs Silencing Suppression Activity Partially Overlaps that of HC-Pro

**Figure 5:**
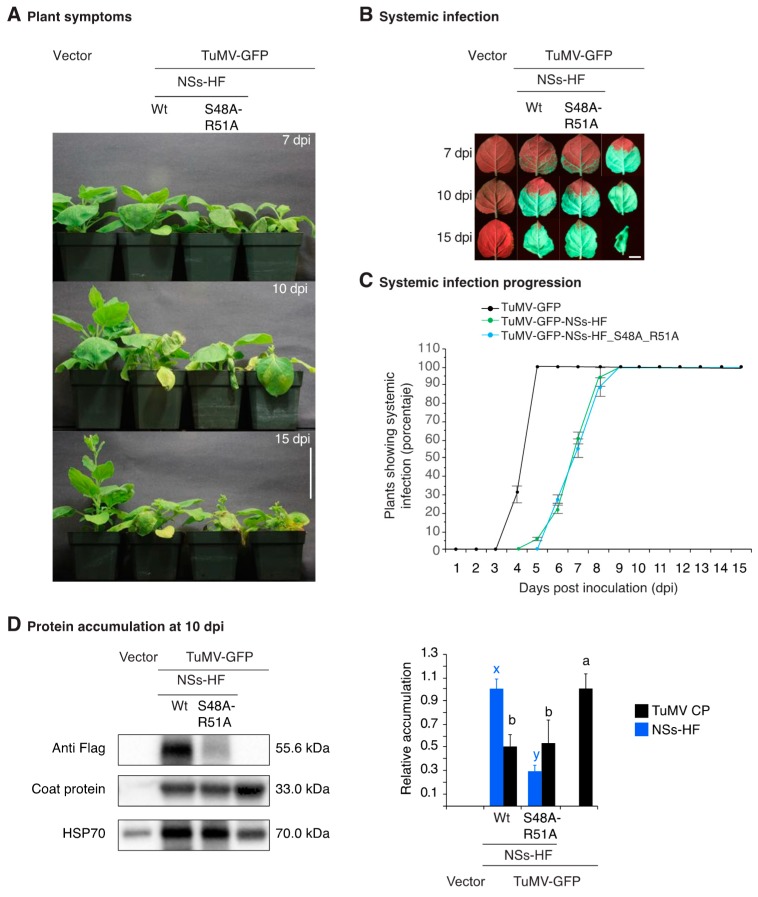
Symptoms, virus distribution and accumulation in noninoculated leaves of *N. benthamiana*. Plants were inoculated by agroinfiltration with empty vector, TuMV-GFP, or TuMV-GFP derivative expressing active or suppressor-deficient NSs-HF. (**A**) Representative plant symptoms at 7, 10 and 15 dpi. Scale bar: 7.5 cm. (**B**) Noninoculated leaves from plants in (**A**) showing representative distribution of virus infection determined by GFP fluorescence under UV light. Scale bar: 2 cm. (**C**) Proportion (%) of plants showing systemic infection as determined by UV illumination. Points represent average (±standard error) of three repetitions, each consisting of 18 plants per treatment. (**D**) Virus accumulation in systemically infected leaves at 10 dpi. Representative western blots showing NS-HF and coat protein accumulation in the same sample. HSP70 was used as a loading control. The histogram shows average (±standard error) coat protein and NSs-HF accumulation (four biological replicates) relative to TuMV-GFP or to wt NSs-HF, respectively. Bars with the same letter do not significantly differ (Tukey’s test with α = 0.05).

**Figure 6:**
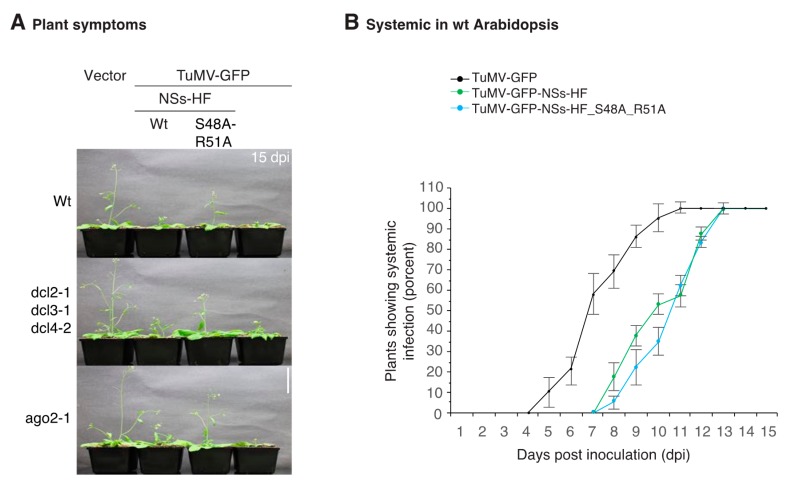
Systemic movement and symptoms in *A. thaliana* (Arabidopsis) plants. Wt, dcl2-1 dcl3-1 dcl4-2 triple mutants or ago2-1 single mutant plants were inoculated by Agroinfiltration with empty vector, TuMV-GFP expressing active or suppressor-deficient NSs-HF, or with TuMV-GFP. (**A**) Representative symptoms in Arabidopsis plants at 15 dpi. Scale bar: 6 cm. (**B**) Proportion (%) of plants showing systemic infection of inflorescence. Points represent average (±standard error) of three repetitions, each consisting of 18 plants per treatment.

In TuMV, HC-Pro and VPg are RNA silencing suppressors and determinants of symptom development [2,21]. The results described above showed that TSWV NSs is an RNA silencing suppressor that promotes systemic virus movement and symptom development (Figure 2 and Figure 4). To gain insight into the mechanistic activity of NSs, we did an epistasis analysis by expressing both HC-Pro and NSs-HF from the same viral construct. If HC-Pro and NSs have similar mechanistic activity and one suppressor is dominant, their combined effect will be similar to that of a single infection. In the absence of dominance, suppressors could compete for a limiting substrate, interfering with each other, and resulting in mild symptoms. In contrast, if HC-Pro and NSs have different mechanistic activities, their cumulative effect would be synergistic and would cause more severe symptoms than that of a single infection. To test this model, *N. benthamiana* (Figure 5A) and *A. thaliana* (Figure 6A) plants were inoculated with chimeric TuMV-GFP expressing NSs-HF or suppressor-deficient NSsHF-S48A-R51A (Figure 2A). Establishment of systemic infection was monitored for 15 dpi and virus accumulation in systemically infected tissue was determined at 10 dpi. TuMV-GFP expressing NSs-HF or suppressor-deficient NSsHF-S48A-R51A established systemic infection at a similar rate and slower than parental TuMV-GFP in both *N. benthamiana* (Figure 5B,C) and *A. thaliana* (Figure 6) plants. Additionally, symptoms were less severe in plants infected with chimeric TuMV expressing NSs than those caused by parental TuMV-GFP (Figure 5A and Figure 6A). In systemically infected *N. benthamiana* leaves, TuMV-GFP expressing active or suppressor-deficient NSs-HF accumulated to 50% of parental TuMV-GFP (Figure 5D). These differences might be related to the slow movement phenotype caused by NSs when expressed from the TuMV genome (Figure 3D).

Chimeric TuMV-GFP expressing both active HC-Pro and active NSs-HF or suppressor-deficient NSsHF-S48A-R51A established systemic infection of leaves from the base to the top of the leaf (Figure 5B), as described for parental TuMV-GFP (Figure 4C). This is in contrast with the spotted distribution in leaves systemically infected by TuMV-AS9-GFP expressing NSs-HF (Figure 2A and Figure 4C). Accordingly, even in the presence of NSs, spatial distribution of TuMV-GFP in systemically infected tissue was determined by HC-Pro. 

Collectively, these results show that symptoms caused by TuMV-GFP and the spatial distribution of virus infection are similar when expressing HC-Pro in combination with active or suppressor-deficient NSs, suggesting that the mechanisms used to suppress antiviral silencing by HC-Pro and NSs are similar and that HC-Pro is dominant over NSs. 

## 4. Discussion

Transgene silencing suppression activity has been demonstrated for NSs of several orthotospoviruses, including TSWV [33,34], impatiens necrotic spot virus [54], groundnut ringspot virus [33], tomato yellow ring virus [33], capsicum chlorosis virus [55], and groundnut bud necrosis virus [56]. NSs blocks the spread of the transgene silencing signal [33], prevents local antiviral RNA silencing [8,55], is required for persistent infection of and transmission by thrips [36], is required for systemic movement in plants [36], and in mixed infections of iris yellow spot virus and TSWV result in synergism, leading to enhanced virus accumulation and movement in *Datura stramonium* [57]. However, the mechanism of RNA silencing suppression by orthotospoviral NSs and its role in virus infection have not been determined. 

The core genetic components and pathway of plant antiviral RNA silencing have been determined using DNA and positive-strand RNA viruses and experimental model plants such as *N. benthamiana* and *A. thaliana* [3,18,45,49,58,59,60,61]. Less is known about negative-strand and ambisense RNA viruses, mainly due to the lack of genetically tractable hosts and, in the case of orthotospoviruses, due to the lack of infectious clones. To gain insight into the mechanism of RNA silencing suppression by NSs and its effect on virus infection, we used suppressor-deficient TuMV-AS9-GFP, *N. benthamiana*, and *A. thaliana* to create a genetically tractable system. Potato virus X and tobacco mosaic virus have been used as vectors to test viral silencing suppressors [62,63]. These systems, and the TuMV vector described here, might cause unexpected effects. The genetic determinants of antiviral RNA silencing against TuMV have been determined and several *A. thaliana* mutants have been characterized [2,3], which allow for the identification of unexpected effects that are independent from silencing suppression. Indeed, a genetic analysis (Figure 3D) showed that the TuMV vector described here has a slow systemic movement phenotype caused by the NSs insert and that is independent from silencing suppression.

A 6HIS-3xFlag (HF) tag was added at the C terminus of TSWV NSs (Figure 1) and a tagged NSs-HF was placed between NIb and the coat protein in a TuMV-AS9-GFP infectious clone (Figure 2A). The NSs insert was maintained in both *N. benthamiana* and *A. thaliana* during a single infection cycle initiated by agroinfiltration (Figure 2D and Figure 3E). Because both host species are amenable to agroinfiltration, genetic stability of the NSs insert after several passages by mechanical inoculation is not necessary and was not determined.

Although the chimeric virus moved at a lower rate (Figure 2C, Figure 3C, Figure 5C, and Figure 6B), NSs-HF supported the establishment of infection and the systemic movement of suppressor-deficient TuMV-AS9-GFP in *N. benthamiana* and *A. thaliana* (Figure 2B and Figure 3A). In contrast, NSs-HF with inactivating mutations in the RNA-binding domain one failed to promote systemic infection in *N. benthamiana* and wt *A. thaliana* (Figure 2B and Figure 3A). The lack of infection was due to the lack of silencing suppression because the virus harboring NSs-HF with inactivating mutations systemically infected dcl2-1 dcl3-1 dcl4-2 *A. thaliana* triple mutants compromised in antiviral RNA silencing (Figure 3A). 

RNA silencing against TuMV is dependent on DCL4 and DCL2 [3]. Accordingly, the gain in pathogenicity in suppression-deficient TuMV-AS9-GFP was dependent on NSs silencing suppression activity (Figure 2B) to overcome the antiviral defense mediated by DCL4 and DCL2. DCL4 and DCL2 process ds viral RNA to form small virus-derived siRNAs [3,49] that associate with AGO proteins to target single-strand viral RNA [2,11,13,64]. TSWV-infected plants and thrips [65] accumulate TSWV-derived siRNAs [66,67,68]. Accordingly, NSs does not prevent the biogenesis of virus-derived siRNAs. However, TSWV isolates harboring a suppressor-defective NSs protein accumulated virus-derived siRNAs to higher levels than isolates harboring a functional NSs, and the virus-derived siRNAs had a different genomic distribution [68]. These observations suggest that NSs acts downstream of virus-derived formation by DCL proteins, possibly at the association with AGO proteins, RNA silencing amplification, or both.

Systemic infection of plants by RNA viruses is restricted in a spatial manner by AGO and RDR proteins [2,3,11]. AGO2 and AGO5 restrict infection in leaves, while AGO1 and AGO10 protect inflorescence from systemic infection by TuMV [2]. Similarly, the redundant activity of RDR1 and RDR6 protect inflorescence from systemic infection by suppressor-deficient TuMV-AS9-GFP [3]. Accordingly, infection of rdr1-1, rdr6-15, and ago2-1 single-mutant plants by suppressor-deficient TuMV-AS9-GFP is restricted to leaves while inflorescences are not infected [2]. However, TuMV-AS9-GFP expressing NSs-HF established systemic infection of inflorescence in wt *A. thaliana* plants and in rdr1-1, rdr6-15, and ago2-1 single-mutant plants (Table 1 and Table 2). Thus, NSs interferes with the antiviral activity of AGO and RDR proteins. 

In addition to HC-Pro, in TuMV, VPg is a suppressor of RNA silencing that causes degradation of SGS3 and RD6, which are involved in RNA silencing amplification [21]. In TuMV-AS9-GFP, VPg is not enough to promote infection of *N. benthamiana* or wt *A. thaliana* [3]. When expressed in cis from TuMV-AS9-GFP, NSs restored pathogenicity and systemic movement both in *N. benthamiana* and in *A. thaliana* (Figure 2 and Figure 3). Accordingly, NSs can functionally replace potyviral HC-Pro. 

In plants infected by TuMV-AS9-GFP expressing NSs, virus special distribution co-localized with chlorotic spots typical of TSWV infection symptoms (Figure 4), instead of those induced by TuMV (Figure 4), suggesting that NSs is a symptom determinant. Consistent with these observations, transgenic plants expressing groundnut bud necrosis virus (GBNV) NSs showed symptoms similar to those infected by GBNV [56]. Symptoms in plants infected by TuMV-GFP expressing NSs resembled symptoms induced by TuMV (Figure 5A), and systemic infection followed a pattern similar to that of TuMV-GFP (Figure 5B). Accordingly, HC-Pro is dominant over NSs. 

Potyviral HC-Pro suppresses antiviral RNA silencing by sequestering small RNAs and preventing their association with AGO proteins [2,17], while CMV 2a targets the initiation step [9]. Consistent with this model, potyviral HC-Pro reactivated expression of a GUS transgene [69], and transgenic GFP silenced plant regained GFP expression upon infection with TSWV or PVY while plants infected with CMV did not regain GFP expression [35]. NSs of TSWV, INSV, GRSV, and TYRV bind small interfering RNAs and miRNA/miRNA* duplexes in vitro [54]. Similarly, potyviral HC-Pro binds several miRNAs and miRNAs* [2,15,16,17]. These observations suggest that NSs suppresses RNA silencing by a mechanism that includes binding of virus-derived siRNAs and host siRNAs. This model is consistent with the presence of three RNA-binding domains in NSs [38].

## 5. Conclusions 

A genetically tractable system was developed to determine the role of TSWV NSs in silencing suppression, pathogenicity, and virus systemic movement. This system is based on a potyvirus infectious clone, *N. benthamiana*, and *A. thaliana*, and is amenable to modifications to study any protein of interest. Using this system, a genetic analysis showed that NSs can functionally replace potyviral HC-Pro, and is genetically stable when expressed in cis from TuMV. NSs suppresses an antiviral defense mechanism that is dependent on DCL, RDR, and AGO proteins to condition virus susceptibility, promote virus infection, systemic movement, and symptom development.

## Figures and Tables

**Table 1:** Systemic infection of *Nicotiana benthamiana* and *Arabidopsis thaliana* by chimeric TuMV-AS9-GFP expressing active or suppressor-deficient NSs-HF ^1^.

Host and Virus	Plants Inoculated	Plants Systemically Infected			
		5 dpi	7 dpi	10 dpi	15 dpi
*Nicotiana benthamiana*					
Empty vector	36	0	0	0	0
TuMV-AS9-GFP	36	0	0	0	0
TuMV-AS9-GFP-NSs-HF	36	2	18	31	36
TuMV-AS9-GFP-NSs-S48A-R51A-HF	36	0	0	0	0
TuMV-GFP	36	36	36	36	36
Wt *Arabidopsis thaliana* (Col-0) ^2^					
Empty vector	36	0	0	0	0
TuMV-AS9-GFP	36	0	0	0	0
TuMV-AS9-GFP-NSs-HF	36	0	0	5	36
TuMV-AS9-GFP-NSs-S48A-R51A-HF	36	0	0	0	0
TuMV-GFP	36	4	23	34	36
*A. thaliana* dcl2-1 dcl3-1 dcl4-2 triple mutant ^2^					
Empty vector	36	0	0	0	0
TuMV-AS9-GFP	36	0	16	32	36
TuMV-AS9-GFP-NSs-HF	36	0	0	15	36
TuMV-AS9-GFP-NSs-S48A-R51A-HF	36	0	0	13	36
TuMV-GFP	36	0	14	32	36

^1^ Counts from three independent experiments were pooled. Establishment of systemic infection was monitored by GFP fluorescence under UV illumination. ^2^ *A. thaliana* plants showing systemic GFP in inflorescence.

**Table 2:** Systemic infection of selected *A. thaliana* mutant plants by chimeric TuMV-AS9-GFP expressing active or suppressor-deficient NSs-HF ^1^.

Host and Virus	Plants Inoculated	Plants Systemically Infected at 15 dpi ^2^			
		Cauline Leaves		Inflorescence	
		Number	Percent	Number	Percent
*A. thaliana* ago2-1 single mutant ^2^					
Empty vector	36	0	0	0	0
TuMV-AS9-GFP	36	36	100	0	0
TuMV-AS9-GFP-NSs-HF	36	36	100	36	100
TuMV-AS9-GFP-NSs-S48A-R51A-HF	36	0	0	0	0
TuMV-GFP	36	36	100	36	100
*A. thaliana* rdr1-1 single mutant ^3^					
Empty vector	36	0	0	0	0
TuMV-AS9-GFP	36	36	100	0	0
TuMV-AS9-GFP-NSs-HF	36	36	100	36	100
TuMV-AS9-GFP-NSs-S48A-R51A-HF	36	0	0	0	0
TuMV-GFP	36	36	100	36	100
*A. thaliana* rdr6-15 single mutant ^3^					
Empty vector	36	0	0	0	0
TuMV-AS9-GFP	36	36	100	0	0
TuMV-AS9-GFP-NSs-HF	36	28	78	28	78
TuMV-AS9-GFP-NSs-S48A-R51A-HF	36	0	0	0	0
TuMV-GFP	36	36	100	36	100

^1^ *A. thaliana* plants showing systemic GFP in cauline leaves or inflorescence as determined by UV illumination. ^2^ Counts from three independent experiments were pooled. ^3^ Counts from two independent experiments were pooled.
